# Current ADC Linker Chemistry

**DOI:** 10.1007/s11095-015-1657-7

**Published:** 2015-03-11

**Authors:** Nareshkumar Jain, Sean W. Smith, Sanjeevani Ghone, Bruce Tomczuk

**Affiliations:** The Chemistry Research Solution, LLC, 360 George Patterson Blvd., Suite 101E, Bristol, Pennsylvania 19007 USA

**Keywords:** ADC, ADC clinical candidates, antibody-drug conjugates, bioconjugates, linker chemistry

## Abstract

The list of ADCs in the clinic continues to grow, bolstered by the success of first two marketed ADCs: ADCETRIS® and Kadcyla®. Currently, there are 40 ADCs in various phases of clinical development. However, only 34 of these have published their structures. Of the 34 disclosed structures, 24 of them use a linkage to the thiol of cysteines on the monoclonal antibody. The remaining 10 candidates utilize chemistry to surface lysines of the antibody. Due to the inherent heterogeneity of conjugation to the multiple lysines or cysteines found in mAbs, significant research efforts are now being directed toward the production of discrete, homogeneous ADC products, via site-specific conjugation. These site-specific conjugations may involve genetic engineering of the mAb to introduce discrete, available cysteines or non-natural amino acids with an orthogonally-reactive functional group handle such as an aldehyde, ketone, azido, or alkynyl tag. These site-specific approaches not only increase the homogeneity of ADCs but also enable novel bio-orthogonal chemistries that utilize reactive moieties other than thiol or amine. This broadens the diversity of linkers that can be utilized which will lead to better linker design in future generations of ADCs.

**Electronic supplementary material:**

The online version of this article (doi:10.1007/s11095-015-1657-7) contains supplementary material, which is available to authorized users.

Current antibody-drug conjugates (ADC) are approaching the realization of the magic bullet theory first proposed by Paul Ehrlich over a century ago, in which he reasoned that if a compound could be made that selectively targeted a disease-causing organism, then a toxin for that organism could be delivered along with the agent of selectivity (1). In its simplest form, ADCs are comprised of an antibody to which is attached a cytotoxic agent through a linker (Fig. 1). The antibodies are generally fully humanized monoclonal antibodies (mAbs) which have high selectivity for tumor-associated antigens, long circulating half-lives, and little to no immunogenicity. Thus, mAbs provide an ideal delivery platform for selective targeting of tumor cells. If combined with cytotoxic agents that can be released within the tumor cells once they have been delivered by the selective antibodies, then the eradication of the tumor cell can be realized while sparing normal cells which have not been targeted by the antibody—thus providing a magic bullet. However, the realization of this theory has taken decades to implement and is still a work in progress.

**Fig. 1:**
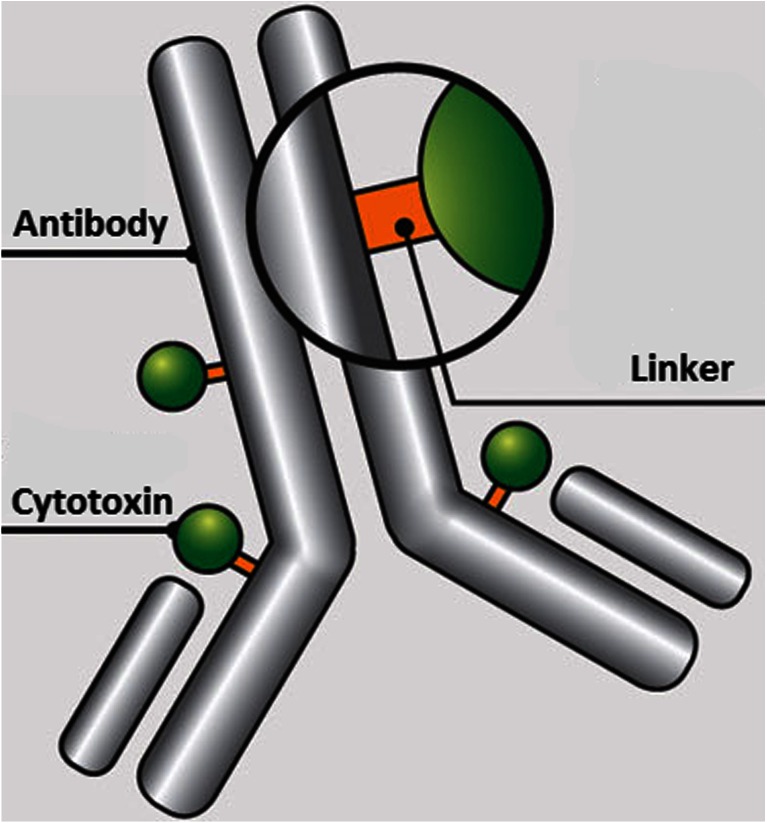
Schematic of an ADC containing an antibody onto which is covalently attached a linker which in turn is covalently attached to a cytotoxin.

The mechanism of action of a successful ADC is depicted in Fig. 2. After being introduced into the plasma (step 1), the ADC recognizes an antigen on the cell surface (step 2). The ADC-antigen complex then undergoes endocytosis, termed internalization (step 3). Once inside the tumor cell, the ADC-antigen complex is fused with the endosome which breaks up the complex for antigen recycling and transports the ADC to the lysosome. Finally, the ADC undergoes various lysosomal degradations to release the cytotoxic drug (step 4), which then binds to its target. The majority of payloads cause cell death or apoptosis via either DNA intercalation or through binding to microtubulins (step 5). Thus, the ideal tumor antigen must be localized on the cell surface in order to allow efficient ADC binding. In addition, the antigen should demonstrate restricted expression on tumor cells, i.e., the antigen should be predominately expressed on tumor cells with minimal expression on normal cells. An important factor for the therapeutic index is the antigen density since a small number of antigenic sites would pose a problem for efficient ADC targeting, internalization and delivery. It has been estimated that the delivery of a lethal quantity of a tubulin-acting payload into a tumor cell may be difficult to achieve below ~10,000 antigen proteins per cell (2). For the antibody, it must be able to bind to tumor-associated antigens with high specificity and high affinity. In addition, the antibody should be non-immunogenic, an issue that has been minimized with chimeric and fully-humanized monoclonal antibodies (3). The ideal payload, or cytotoxin, needs to have high potency against the specific tumor type since it has been estimated that only 1–2% of the administered drug reached the intracellular target (e.g., tumoral DNA or microtubules) (4). The tubulin-binding cytotoxins, such as the maytansinoids or the auristatins, have *in vitro* cytotoxic potencies in the picomolar range (10^−12^ M). What has become clear is that every component of an ADC must be optimized in order to fully realize the goal of a targeted therapy with improved efficacy and tolerability.

**Fig. 2:**
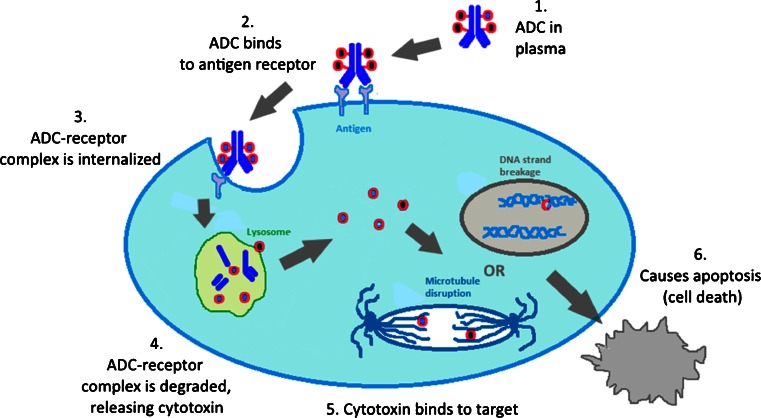
Mechanism of action of ADCs: The antibody portion of an ADC hones onto a cell-surface antigen that is ideally specific to a cancer cell. Upon binding, the ADC-antigen protein complex becomes internalized into the cancer cell. When the complex is degraded, it releases the cytotoxin which then binds to its target to cause cancer cell apoptosis.

Recently, there has been a great deal of discussion in the literature regarding ADCs, much of which has focused on the biological characteristics of this class of therapeutics (5–7). However, the focus of this review is from a chemistry perspective of the linker.

## The Linker

The linker, which is the highlight of this review, needs to possess a number of key attributes, including the requirement to be stable in plasma after drug administration for an extended period of time such that the ADC can localize to tumor cells. This stability prevents the premature release of the cytotoxic payload, which would indiscriminately damage tissue of all kinds, thereby lowering the therapeutic index of the ADC. Upon internalization, the ADC should liberate the payload such that the payload can bind to its target. In addition to these two basic functions, linkers can have a profound effect on the physico-chemical properties of the ADC. In particular, most of the cytotoxic payloads are hydrophobic in nature. Thus, linking them to the mAb with an additional hydrophobic moiety can create problems due to aggregation. ADC aggregates are insoluble and often limit achievable drug loading onto the antibody. It has been conjectured that ADC aggregates are sequestered in the liver, leading to hepatotoxicity (8). Lastly, protein aggregation of biologics, in general, has been linked to increased immunogenicity (9). Due to these safety concerns, aggregation needs to be monitored over time in ADC products to establish a safe shelf-life. In addition to aggregation problems, the hydrophobic characteristic of the majority of cytotoxic compounds in ADCs causes them to be good substrates for multidrug resistance (MDR) transporters. These transporters are responsible for effluxing cytotoxins out of the tumor cells, thus compromising efficacy. However, Kovtun *et al.* has shown that using a hydrophilic linker with maytansinoids produced a hydrophilic metabolite of DM1 which was not an MDR substrate (10). This hydrophilic-linked DM1 ADC was markedly more effective in an MDR-1-expressing human xenograft tumor than the MCC-DM1 ADC.

## Linker Chemistry-Linkage to Antibody

**Fig. 3:**
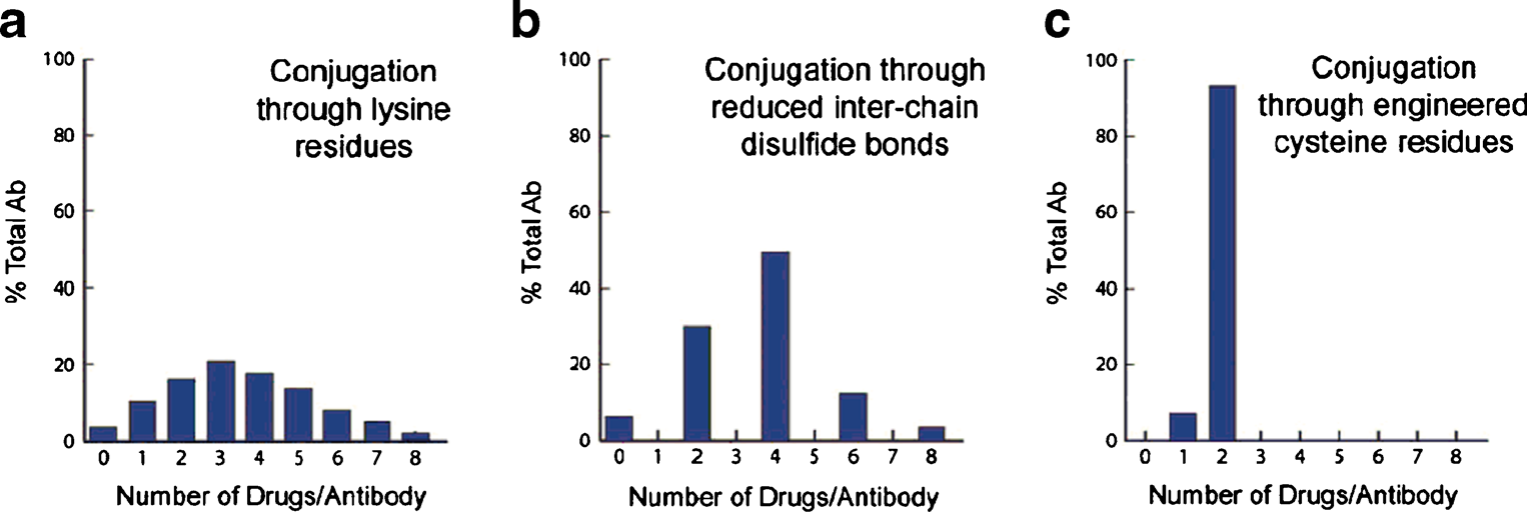
(11) Heterogeneity observed through different means of antibody-payload conjugation. Panel A depicts conjugation through lysines. Since approximately 30 of the 80 lysines on an IgG1 are available for conjugation, several species containing different drug-antibody ratios (DAR) are formed. Panel B depicts the reduced hetereogeneity of conjugation through reduced disulfides. Panel C indicates that a DAR of 1 or 2 is obtained via attachment of the cytotoxin to engineered cysteines

Initially, the chemistry available for linkage to the polypeptide of antibodies was limited to those natural amino acids that have side-chains with available nucleophilic groups such as the ε-NH_2_ of lysine and the sulfhydryl SH group of cysteine. Conjugation via lysine can involve a two-step process in which a linker is attached to the mAb (mAb-L) followed by a second chemical reaction of the linker with the cytotoxic drug molecule or can involve a one-step reaction with a pre-formed L-D to form the mAb-L-D. A typical IgG1 antibody has roughly 90 lysine residues, of which approximately 30 can be modified under forcing conditions (http://www.lifetechnologies.com/us/en/home/references/molecular-probes-the-handbook.html). Random conjugation utilizing lysine generates a heterogeneous mixture of conjugate species (11). The mixture is generally characterized by the average drug-to-antibody ratio, or DAR. Typically, a DAR of 3–4 is targeted, as illustrated in Panel A of Fig. 3. However, it must be noted that this heterogeneity is two-fold since the drug can be conjugated to any number of the approximate 30 available lysines. This produces a heterogeneous mixture of several subspecies some of which may have altered antigen-binding properties leading to suboptimal potency, solubility, stability, and/or pharmacokinetics.

Conjugation to the cysteines of an antibody differs in that the cysteines are involved in intrachain and interchain disulfide bridges. Under controlled reduction conditions, the interchain disulfide bonds can be reduced to create sulfhydryls (Cys-SH) available for conjugation, while leaving the intrachain disulfides intact. Since there are only 4 interchain disulfide bridges in an IgG1, there are only 8 possible conjugation sites, thus conjugation via cysteine generates markedly lower heterogeneity compared to lysine-based conjugation and produces a mixture composed of even-numbered cytotoxin-loaded species as depicted in Panel B of Fig. 3. Still, the heterogeneity inherent with both of these types of conjugation can cause challenges during the scale-up and production of ADCs due to potential batch-to-batch variability.

A solution to the inherent heterogeneity of antibody-drug conjugates has been to introduce engineered cysteines into the sequence of the antibody such that only 1 or 2 conjugates can be formed as demonstrated in Panel C. This work, pioneered by Junutula *et al.*, involved phage display for selection of reactive thiols that do not alter antigen binding or function (12). These engineered thio antibodies were termed THIOMAB conjugates. The anti-MUC16 THIOMAB was shown to be as efficacious as the traditional conjugate with approximately half the drug dose and was better tolerated in rat and monkey toxicity studies.

## Release of Cytotoxin

There are two families of linkers, cleavable and non-cleavable. Cleavable linkers utilize an inherent property of a tumor cell for selective release of the cytotoxin from the ADC. There are three commonly used mechanisms: 1) protease-sensitivity, 2) pH-sensitivity, and 3) glutathione-sensitivity. The protease-sensitive strategy utilizes the dominant proteases found in a tumor cell lysosome for recognition and cleavage of a specific peptide sequence in the linker. Dubowchik and Firestone *et al.* pioneered the discovery of the valine-citrulline (vc) dipeptide as a intracellular cleavage mechanism by cathepsin B (13). The acid-sensitivity strategy takes advantage of the lower pH in the endosomal (pH = 5–6) and lysosomal (pH = 4.8) compartments, as compared to the cytosol (pH = 7.4), to trigger hydrolysis of an acid labile group within the linker, such as a hydrazone (14). The third release strategy exploits the higher concentrations of intracellular glutathione than in the plasma. Thus, linkers containing a disulfide bridge release the cytotoxin upon reduction by glutathione. Non-cleavable linkers contain no obvious release mechanism. This strategy relies on the complete degradation of the antibody after internalization of the ADC. As a consequence of this degradation, the linker will carry an amino acid from the antibody. Below, linkers employing these various release strategies will be discussed for each cytotoxin class since the few linker-drug motifs that have been successful have repeatedly been used for other antibodies.

## Linker-Cytotoxin Motifs

The pioneering ADC, Mylotarg® (Gemtuzumab ozogamicin), was approved in 2000. The construct of Mylotarg® consisted of a relatively labile hydrazone linker with the cytotoxin, calicheamicin. However, a confirmatory post-approval study was stopped early due to safety concerns and a lack of clinical benefit. It is believed that the heterogeneous nature of the conjugate and hydrazone linker instability played a role in the safety of Mylotarg® and it was voluntarily withdrawn from the market in 2010. Interestingly, there are currently two clinical candidates that utilize hydrazone constructs different from Mylotarg®, the first, CMC-544 (Inotuzumab ozogamicin), is calicheamicin-based and the second, milatuzumab-doxorubicin, is doxorubicin derived. In the last several years, two ADCs have been approved; this includes ADCETRIS® (Brentuximab vedotin) in 2011 for relapsed Hodgkin lymphoma and Kadcyla® (Trastuzumab emtansine) in 2013 for HER2^+^ breast cancer. ADCETRIS® utilizes a protease-sensitive linker-drug motif discussed later under auristatins and Kadcyla® uses a non-cleavable linker-drug motif to be discussed under the maytansines section. In addition, there are 40 ADCs at various stages of clinical development as of December 2014 (see Electronic supplementary material).

## Cysteine Linkers

**Fig. 4:**
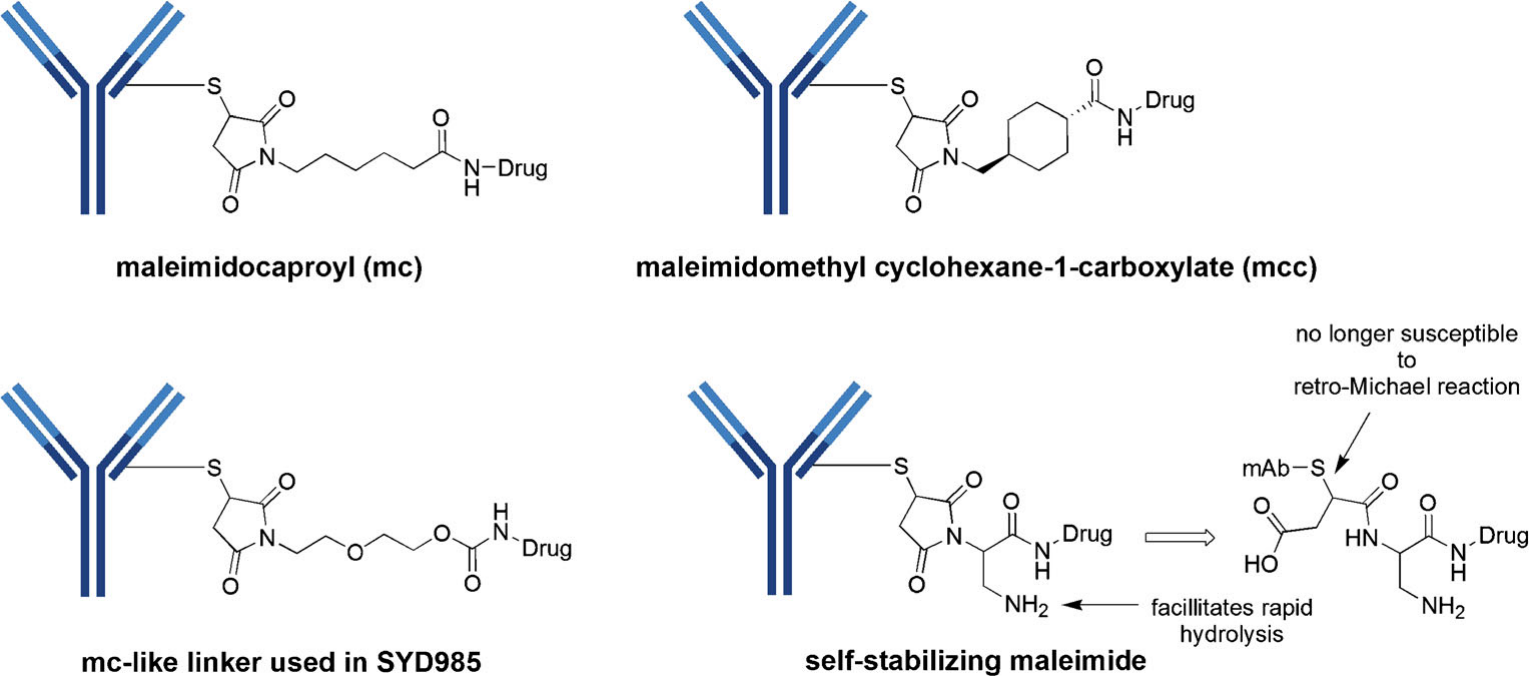
Maleimide chemistry has been the mainstay for linkage to cysteines. Two common variants are the maleimidocaproyl (mc) and maleimidomethyl cyclohexane-1-carboxylate (mcc). Also illustrated is the self-stabilizing maleimide construct designed to prevent early release of the cytotoxin via retro-Michael reaction

Of the 40 ADCs that are in clinical trials, only 34 have disclosed the structures. The majority of these structures (24 out of 34) utilize attachment of the linker-drug directly to cysteine of the antibody. In fact, all of these take advantage of the excellent reactivity of maleimide with sulfhydryl groups. There are two common maleimide-type linkers: maleimidocaproyl (mc) and maleimidomethyl cyclohexane-1-carboxylate (abbreviated “mcc” in this review) (Fig. 4). All of the auristatin-containing ADCs, both monomethyl auristatin E (MMAE) and monomethyl auristatin F (MMAF), utilize the “mc” linkage to the antibody. One negative aspect of utilizing maleimide chemistry for cysteine linkage has been its chemical instability in plasma, including a retro-Michael reaction which results in premature loss of the drug-linker from the ADC. Senter *et al.* realized that hydrolysis of the thiosuccinimide ring prevented this elimination. Thus, this group incorporated a basic amino functionality adjacent to the maleimide to induce rapid hydrolysis at neutral pH. These self-hydrolyzing maleimide-containing ADCs have shown improved potency (15).

## Cysteine-Linker-Auristatin Motifs

**Fig. 5:**
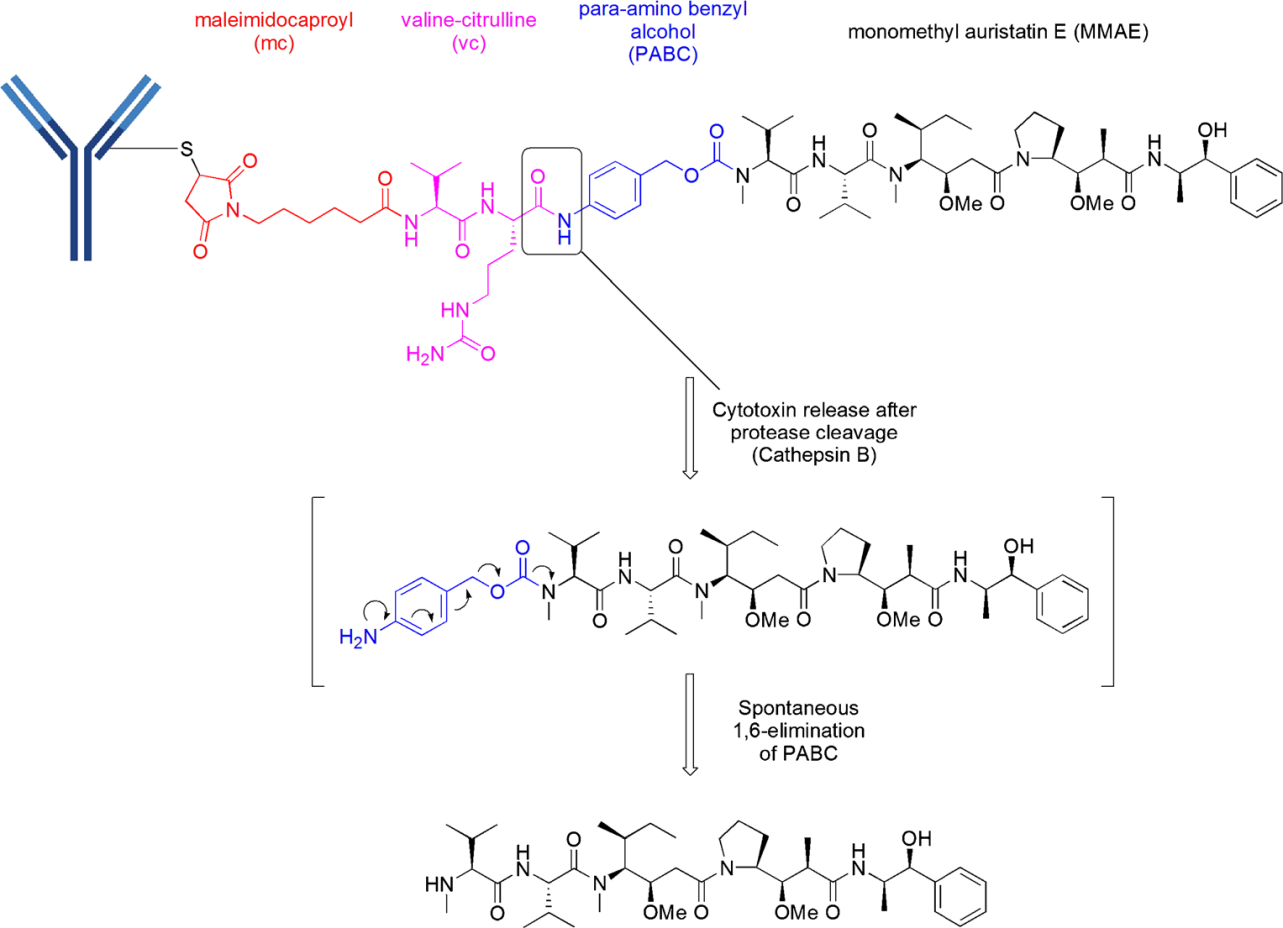
A common structure for MMAE linkage has been the use of “mc” attached to a protease recognition sequence of valine-citrulline (vc), which in turn is attached to a para-amino benzyl alcohol (self-immolative moiety)

Thirteen of the 18 disclosed auristatin-containing structures utilize the “vc-MMAE” linker-drug combination (Table I). Although abbreviated vc-MMAE, the construct is actually better described as mc-vc-PABC-MMAE. This linker-drug, mc-vc-PABC-MMAE, is conjugated to cysteine after reduction of the interchain cysteines of the antibody, producing an ADC with a DAR of 4. The construct utilizes a maleimidocaproyl (mc) spacer, a protease-sensitive dipeptide, valine-citrulline (vc), a self-immolative spacer, para-amino benzyloxycarbonyl (PABC), and the antimitotic agent, monomethyl auristatin E (MMAE) as illustrated in Fig. 5. The purpose of the mc spacer is to provide enough room so that the vc group can be recognized by cathepsin B, which cleaves the citrulline-PABC amide bond. The resultant PABC-substituted MMAE is not a stable intermediate and spontaneously undergoes a 1,6-elimination with a loss of *p*-iminoquinone methide and carbon dioxide (self-immolation) leaving MMAE as the product. The optimization of this design was an evolutionary process which entailed much experimentation (16). This design was successfully used in ADCETRIS®, an anti-CD30-vc-MMAE construct, for relapsed Hodgkin lymphoma.

**Table I:** Clinical ADCs utilizing “vc-MMAE”

Ph	Originator	Licensee (L)/collaborator (C)	Common name	Specificity target name	Targeted disease
3	Seattle Genetics	Celldex (L)	CDX-011	anti-GPNMB	breast cancer
2	Seattle Genetics	Progenics		anti-PSMA	prostate
2	Seattle Genetics	Genentech	RG-7596	anti-CD79b	NHL
2	Seattle Genetics	Genentech	RG-7593	anti-CD22	NHL
2	Seattle Genetics	Genentech	RG-7599	anti-NaPi2b	ovarian cancer
1	Seattle Genetics		SGN-LIV1A	anti-Liv1	LIV1^+^ breast cancer
1	Seattle Genetics	Agensys(C)	ASG-22ME	Nectin-4	solid tumors
1	Seattle Genetics	Agensys(C)	ASG-15ME	SLTRK6	bladder cancer
1	Seattle Genetics	Genentech	RG-7450	anti-STEAP1	prostate
1	Seattle Genetics	Genentech	RG-7458	anti-MUC16	ovarian
1	Agensys		AGS67E	anti-CD37	NHL, CLL, AML
1	Seattle Genetics	GenMab	HuMax-TF	anti-TF	solid tumors
1	Seattle Genetics	Takeda	MLN-0264	anti-GCC	gastrointestinal cancer

**Fig. 6:**
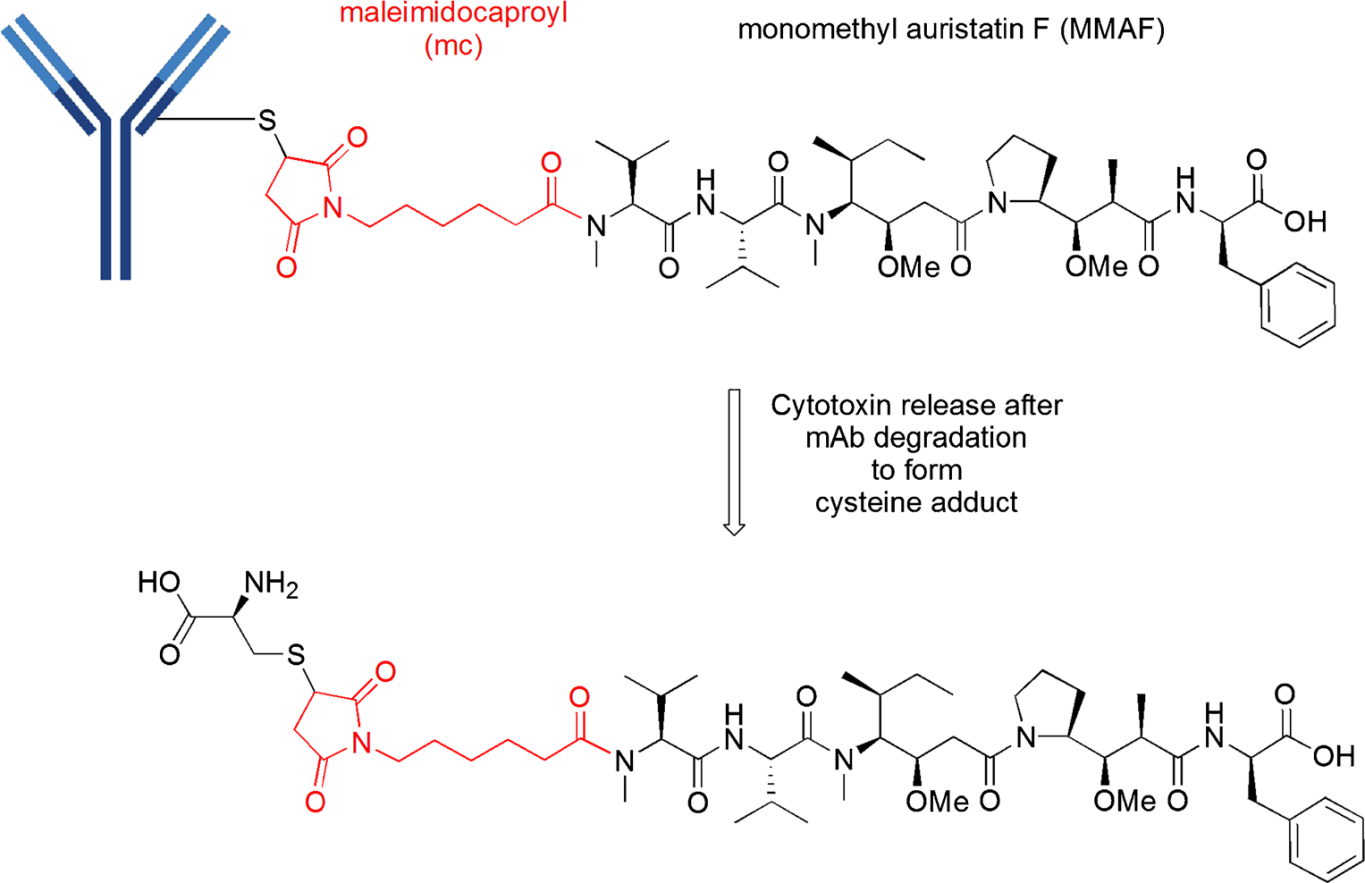
Construct of a mc-MMAF ADC

An analog of MMAE in which the terminal ephedrine group in replaced by phenylalanine, termed monomethyl auristatin F (MMAF), produced a “vc-MMAF” cAC10 ADC in which the cytotoxicity was >2200-fold more potent than the corresponding free MMAF. This reduced inherent cytotoxicity of the unconjugated, free drug, is presumably due to the charged C-terminal carboxylate of phenylalanine that impairs intracellular access in normal cells. Thus, this provides the rationale for the observed larger therapeutic window for cAC10-vc-MMAF than the corresponding cAC10-vc-MMAE (17). However, the most unexpected finding was that the mc-vc-PABC linker could be replaced with maleimidocaproyl (mc), which is termed “mc-MMAF”. This particular ADC was equipotent to the one possessing a cleavable linker but without an obvious release mechanism. Using radiolabelling, it was demonstrated that mc-MMAF is released after mAb degradation to produce the cysteine adduct as illustrated in Fig. 6 (17). The therapeutic window for the cAC10-mc-MMAF ADC is at least 3-fold higher than for cAC10-vc-MMAF. Thus, there has been significant effort in utilizing this simple linkage system and there are currently five disclosed mc-MMAF ADCs in the clinic, all in phase 1, as shown in Table II.

**Table II:** Clinical ADCs Utilizing “mc-MMAF”

Ph	Originator	Licensee	Common name	Specificity target name	Targeted disease
1	Agensys		AGS-16M8F/16C3F	anti-AGS-16	RCC
1	Seattle Genetics	Pfizer	PF-06263507	anti-5 T4	solid tumors
1	Seattle Genetics	Abbvie	ABT-414	anti-EGFR	SC tumors & glioblastoma
1	GSK		GSK2857916	anti-BCMA	MM
1	Seattle Genetics		SGN-CD19A	anti-CD19	ALL & B-cell NHL

## Cysteine-Linker-PBD Dimer Motif

**Fig. 7:**
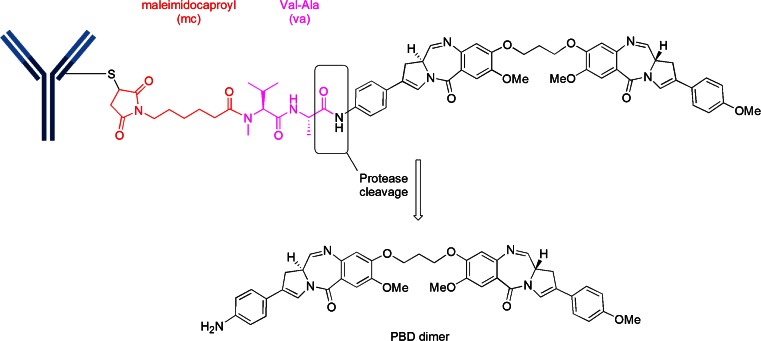
Construct of PBD dimer ADCs. The mc-va-PBD dimer construct is utilized in both clinical ADCs

The pyrrolobenzodiazepines (PBD) are a class of naturally occurring antitumor antibiotics that bind to the minor groove of DNA (18). The dimerization of two PBD units by a suitable spacer dramatically increases the ability of the dimer to cross-link DNA via the N2 of guanine from opposing DNA strands (19). There are two PBD dimer-containing ADCs in Phase 1 clinical trials, both from Seattle Genetics. They are SGN-CD33A which is linked via an engineered-cysteine, termed ec-mAb, to mc and a protease-cleavable, valine-alanine (va) linker to an aniline on the PBD dimer (Fig. 7). SGN-CD33A is being tested in acute myeloid leukemia (AML). The other PBD dimer ADC, SGN-CD70A, is similarly linked to an engineered cysteine and is being tested in non-Hodgkin Lymphoma (NHL) and renal cell carcinoma (RCC). It is noteworthy that both SGN-CD33A and SGN-CD70A represent the first site-specific ADCs to enter clinical trials.

**Table:** 

**Phase**	**Originator**	**Common name**	**Specificity target**	**Target disease**
1	Seattle Genetics	SGN-CD33A	anti-CD33	AML
1	Seattle Genetics	SGN-CD70A	anti-CD70	NHL & RCC

## Cysteine-Linker-Duocarmycin Motif

**Fig. 8:**
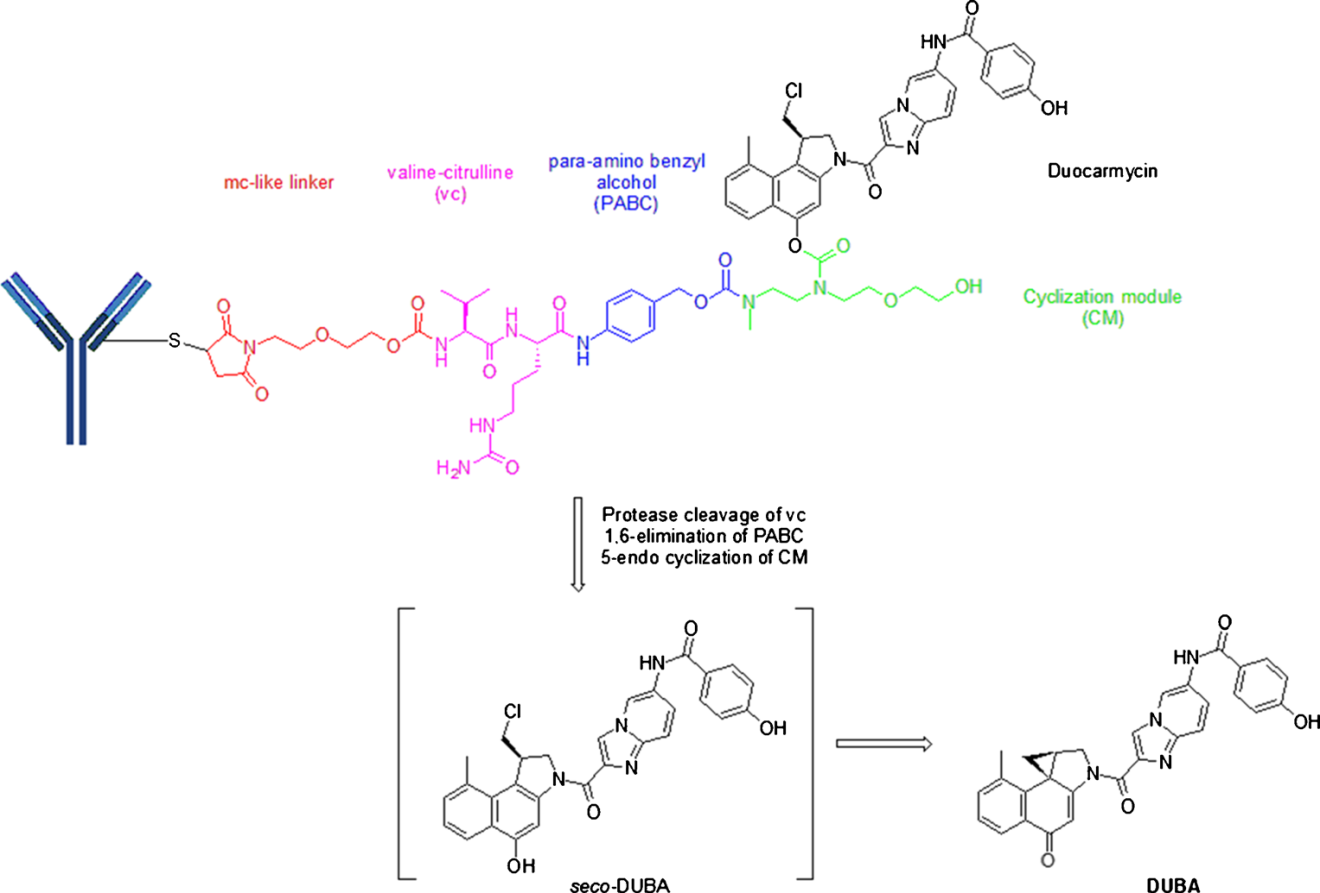
“mc”-vc-PABC-CM-seco-DUBA (SYD985) construct

Duocarmycins, which are DNA alkylating cytotoxins, have been linked via a “mc-like” spacer to partially reduced trastuzumab in SYD-985 (20). The mc is attached to a protease-sensitive linker, valine-citrulline (vc), which is in-turn linked to a PABC and further connected to a cyclization module (CM) before forming a carbamate with the phenolic group of a modified duocarmycin. Figure 8 depicts the presumed release mechanism of DUBA (DUocarmycin-hydroxyBenzamide-Azaindole linker). Protease cleavage followed by self-immolation of the PABC group via 1,6-elimination and of the cyclization module group liberates the free drug. The intermediate *seco*-DUBA is not a stable species and undergoes a rearrangement with the formation of a cyclopropane ring, a reactive, electrophilic construct for DNA alkylation.

**Table:** 

**Phase**	**Originator**	**Common name**	**Specificity target**	**Target disease**
1	Synthon	SYD985	anti-HER2	solid tumors

## Cysteine-Linker-SN38 Motif

**Fig. 9:**
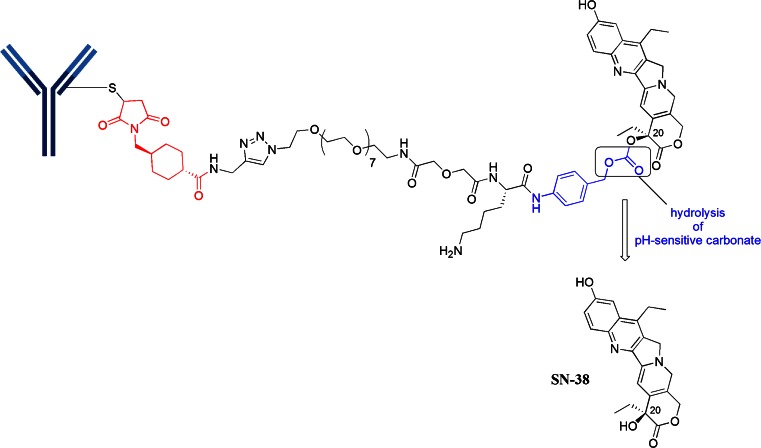
mcc-triazole spacer-PEG7-x-Lys-PABC-SN-38 motif

Immunomedics has been utilizing SN-38, a metabolite of irinotecan (a topoisomerase I inhibitor), as a cytotoxin. SN-38 is about 3 orders of magnitude more potent than irinotecan. SN-38 has been linked to two different mAbs, the resultant ADCs are both in phase 2 clinical trials: IMMU-130 (labetuzumab-SN38, anti-CEACAM5) is being tested in colorectal cancer (CRC) and IMMU-132 (anti-TROP-2) is being pursued in solid tumors (21, 22). Both ADCs use a similar linker construct as shown in Fig. 9. The linker construct consists of mcc-triazole spacer-PEG7-x-lysine-PABC-SN-38. While the linker does not contain a protease-sensitive moiety, it does contain an acid-sensitive carbonate formed with the C-20 hydroxyl group of SN-38. Under lysosomally relevant conditions of pH ~5, this construct has a half-life of 10 h (23). It was also discovered that this C-20 attachment preserves the lactone which is a key feature for bioactivity. The PEG7 spacer provided sufficient solubility. With this particular linker, it was also determined that a high DAR of about 6 molecules of drug per IgG was optimal. The roles of the other parts of the linker are unclear.

**Table:** 

**Phase**	**Originator**	**Common name**	**Specificity target**	**Target disease**
2	Immunomedics	IMMU-130	anti-CEACAM5	CRC
2	Immunomedics	IMMU-132	anti-TROP-2	solid tumors

## Cysteine-Linker-Doxorubicin Motif

**Fig. 10:**
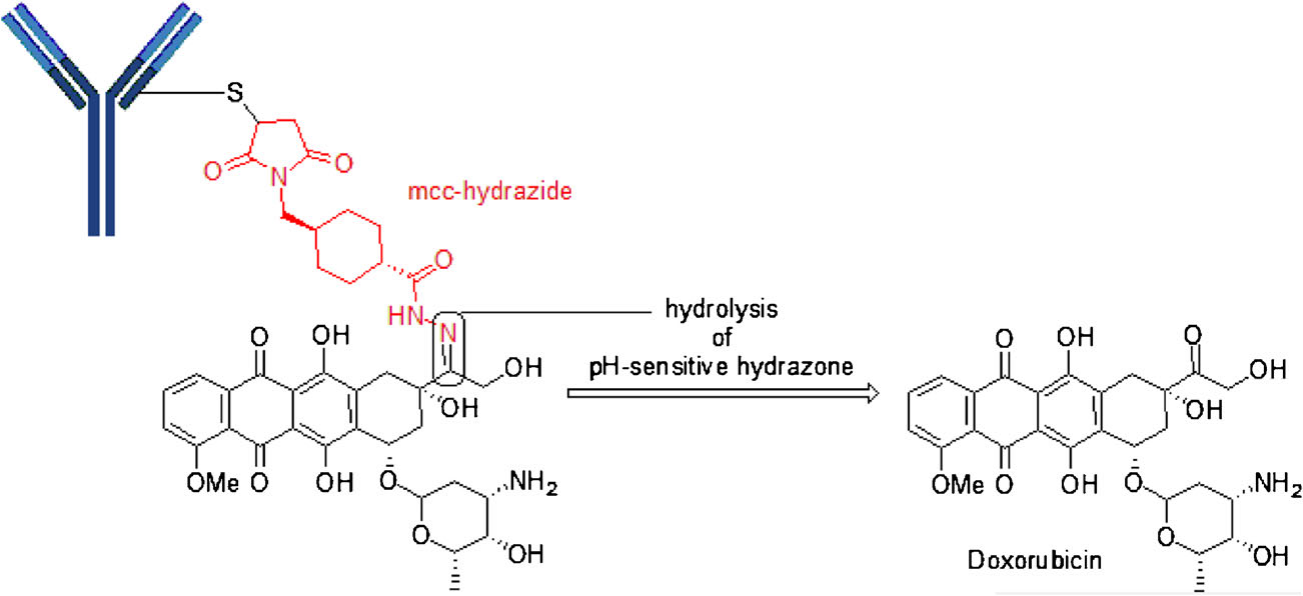
Illustrates the use of mcc in an acyl-hydrazone structure found in Milatuzumab-Doxorubicin

Milatuzumab-doxorubicin (anti-CD74) utilizes a spacer other than mc, while still relying on cysteine-maleimide chemistry. In this motif, a maleimidomethyl cyclohexane-1-carboxyl hydrazide is reacted with doxorubicin to form a mcc-hydrazone linked-doxorubicin which is then reacted with the reduced cysteines (SH) on milatuzumab (Fig. 10). Milatuzumab-doxorubicin is being pursued in Phase 1 studies for NHL and chronic lymphocytic leukemia (CLL). Obviously, the pH-sensitive hydrazone provides the release mechanism for doxorubicin.

**Table:** 

**Phase**	**Originator**	**Common name**	**Specificity target name**	**Targeted disease**
1	Immunomedics	IMMU-110/MEDI-115	anti-CD74	NHL/CLL

## Novel Cysteine Cross-Linkers

**Fig. 11:**
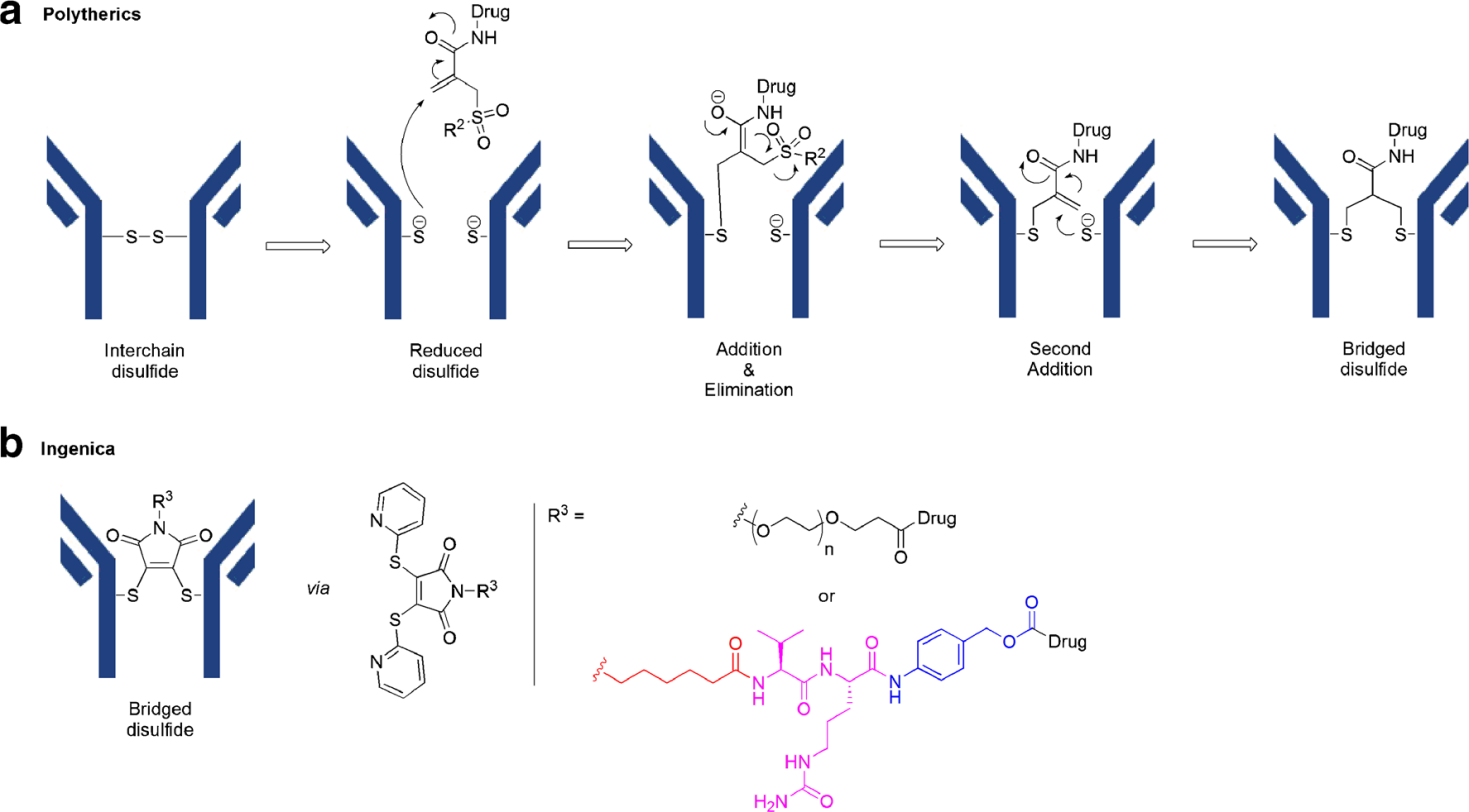
Panel A describes 3-carbon bridge by PolyTherics and Panel B describes 2-carbon bridge by Igenica

As previously mentioned, an IgG1 has 4 interchain disulfides which are reduced to produce reactive sulfhydryl groups. Using the maleimide chemistries outlined above produces conjugation heterogeneity and destabilizes the higher order structure of the IgG due to the loss of these interchain cross-linkages (24). As an alternative, novel strategies are being pursued which allow for rebridging of the reduced disulfide bonds with reagents that carry the cytotoxin. Thus, both sulfhydryl groups derived from a reduced disulfide bond are rebridged by the reagent. PolyTherics has published work using a sulfone reagent which rebridges the disulfide with an intervening 3-carbon bridge as shown in Panel A of Fig. 11 (25). In a similar fashion, Igenica has a bis-reactive agent, a di-thiopyridylmaleimide (DTM), which produces a 2-carbon bridge as shown in Panel B of Fig. 11 (http://adc-summit.com/uploads/files/2463_ADC_/David_Jackson.pdf). Both of these reagents have the potential of producing a homogeneous product with a DAR of 4. However, there are currently no ADC clinical candidates which utilize these reagents.

## Lysine Linkers

**Fig. 12:**
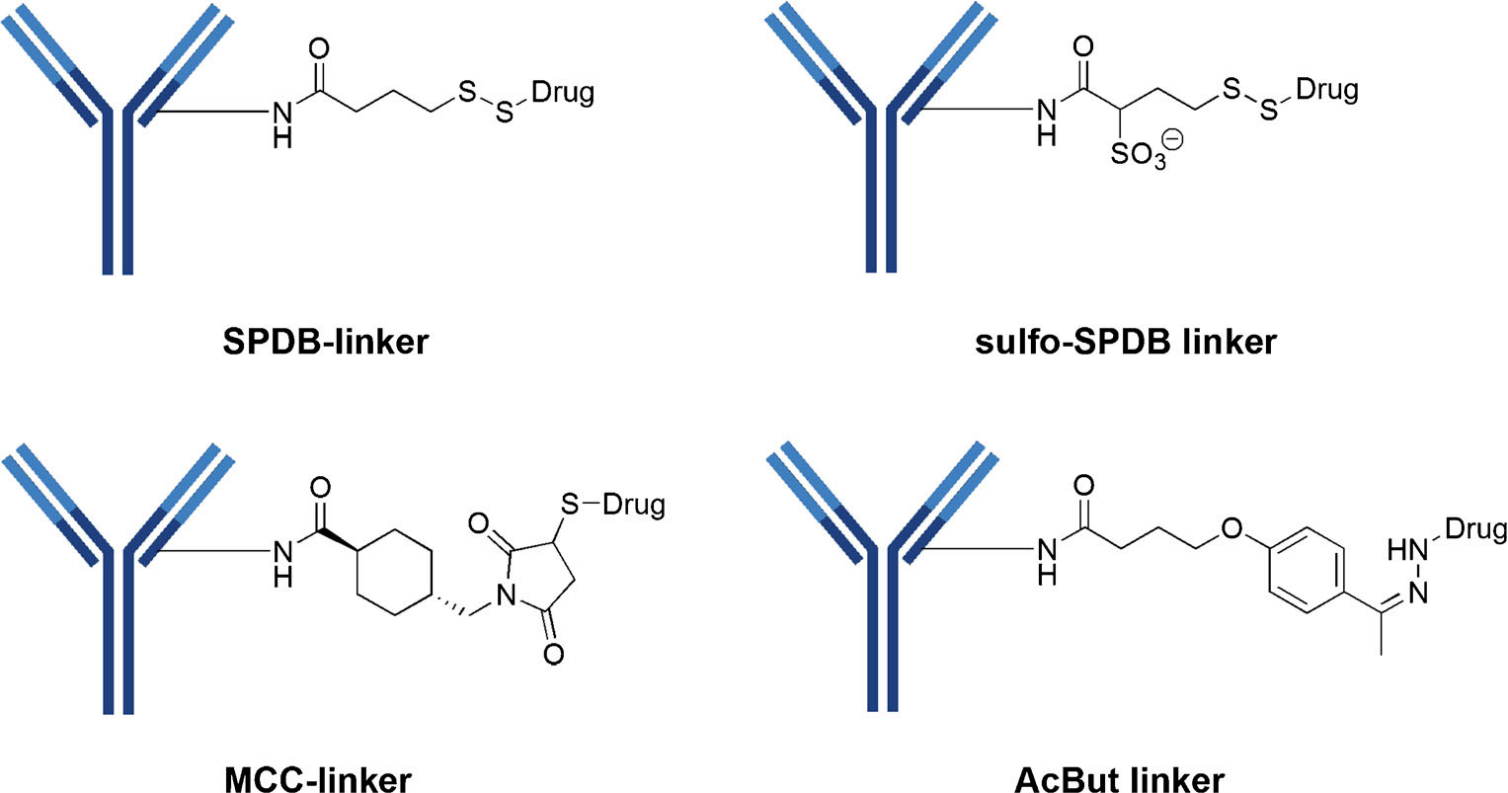
Lysine linkers involve the formation of amides with the ε-amino group of lysine. The most common linkers found in current clinical candidates have been MCC and SPDB, which are both uncharged, lipophilic linkers. Recent research has focused on the introduction of charged, hydrophilic groups, like sulfo-SPDB to minimize aggregation tendencies

Despite the inherent increased heterogeneity, there are 10 clinical candidates that still utilize the reactivity of the ε-amino group of lysine—almost all involve acylation chemistry. In most of these cases, the conjugation is carried out in a two-step fashion. In the first step, a bi-functional reagent (containing amine- and thiol-reactive functional groups) is reacted with available ε-amino groups of lysine (i.e., acylation). In the second step, the cytotoxin-linker (which possesses a reactive sulfhydryl) is attached to the available thiol-reactive group introduced in step one. There are four types of lysine linkers currently used in clinical candidates (Fig. 12): 1) SPDB disulfide, 2) MCC (maleimidomethyl cyclohexane-1-carboxylate), 3) sulfo-SPDB which adds a charged polar group and 4) hydrazone (as used in CMC-544).

## Linker-Maytansinoid Motifs

**Fig. 13:**
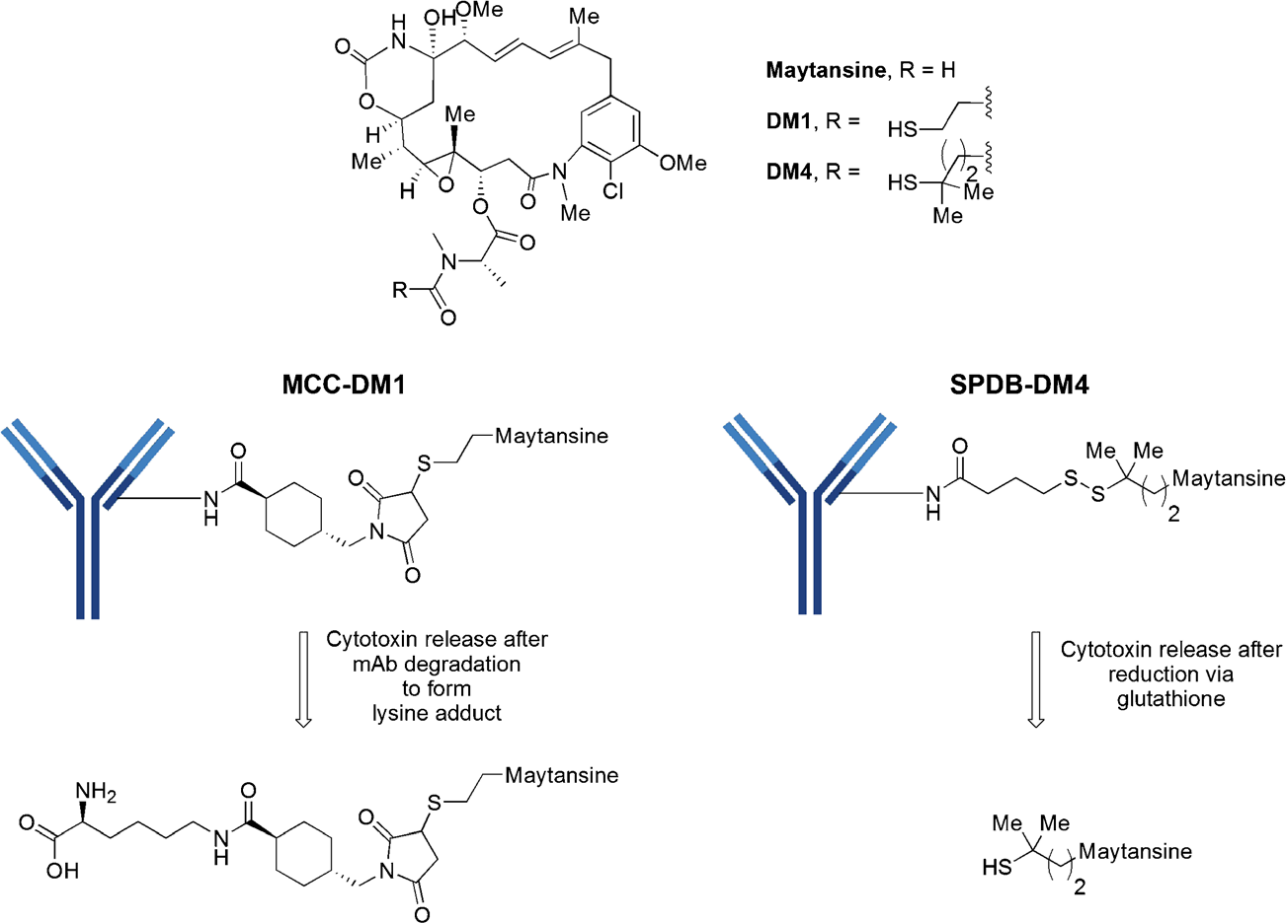
MCC-DM1 and SPDB-DM4 motifs used in the clinical candidates listed in Table IV

The cytotoxic drug class known as maytansines has been modified to contain a reactive sulfhydryl handle. Thus, 9 out of 10 of the lysine-linked clinical ADCs are maytansinoids (Table III). Five of these candidates have a derivative known as DM4 and the remaining four contain a derivative known as DM1. The maytansinoid core of DM1 and DM4 is the same, but the substitution adjacent to the sulfhydryl handle is different, as illustrated in Fig. 13. It is important to note that Kadcyla®, the ADC approved in 2013 for the treatment of Her2^+^ breast cancer, is a MCC-DM1 construct. The conjugation is a two-step process which involves a conjugation of the NHS-activated ester of the bifunctional linker, SMCC, to trastuzumab followed by the reaction of the sulfhydryl from DM1 with the maleimide. The manufacturing process has been standardized to produce a Poisson distribution of 0 to 8 drugs per antibody with a DAR of 3.5. Approximately 70 sites out of 88 total lysines and four N-terminal amines have been identified as participating in the attachment of MCC-DM1 (26).

**Table III:** Clinical ADCs Utilizing MCC-DM1 or SPDB-DM4

Phase	Originator	Licensee (L)/ collaborator(C)	Common name	Specificity target name	Targeted disease
MCC-DM1 ADCs					
1	ImmunoGen		IMGN 289	anti-EGFR	NSCLC, SCCHN, SC NSCLC
1	ImmunoGen		IMGN 529		NHL/CLL
1	ImmunoGen	Amgen	AMG 595	anti-EGFRvIII	glioblastoma
1	ImmunoGen	Amgen	AMG 172	anti-CD27L	
SPDB-DM4 ADCs					
2	ImmunoGen		SAR3419	anti-CD19	diffuse large B-cell lymphoma
2^a^	ImmunoGen		IMGN 853	anti-FRa	
2	ImmunoGen	Biotest	BT-062	anti-CD138	MM
1	ImmunoGen	Sanofi	SAR 566658	anti-CA6	solid tumors
1	ImmunoGen	Bayer	BAY 94-9343	anti-mesothelin	

^a^ IMGN 853 uses sulfo-SPDB linker

It was discovered that the unhindered disulfide-bond of DM1 was too unstable in plasma leading to fast plasma clearance with undetectable levels of ADC by day 3. An increase in local steric hindrance by the addition of alpha-methyl groups next to the disulfide provided the expected increase in plasma stability. The construct possessing alpha *gem*-dimethyl groups on the maytansinoid side (DM4) and one methyl group on the antibody side was as stable as the non-cleavable linker, MCC-DM1 (27). It is known that as the linker becomes more hydrophobic, aggregation become problematic. There have been efforts to develop more soluble linkers for the maytansinoids. Linkers containing the uncharged polyethylene glycol (PEG) spacers and pendant sulfonate groups (such as sulfo-SPDB shown in Fig. 12) have been evaluated. Aside from limiting the formation of aggregates, the polarity of these spacers also alter the interaction of the ADCs with permeability glycoprotein (Pgp) which is a major cause of multi-drug resistance (MDR) by the tumor cells (28). In one study, an ADC using a PEG_4_Mal linker with DM1 was markedly more effective in eradicating MDR-expressing xenograft tumors than the ADC with the MCC-DM1 linker (10). In a similar manner, an ADC using the sulfo-SPDB linker with DM4 was considerably more potent against an MDR expressing cell line with an IC_50_ = 7–20 pM vs >3000 pM for the ADCs with SPDB or MCC linkers (28).

To reiterate, linkers can be classified as cleavable or non-cleavable. Cleavable linkers possess functionalities susceptible to degradation through lysosomal processes (protease-sensitive, acid-sensitive, and reduction-sensitive). ADCs made with non-cleavable linkers, in comparison, have extended plasma half-life which is a desirable attribute. The activity of these non-cleavable linkers in the tumor cells relies on the degradation of the total antibody, ultimately releasing an amino-acid-linker-cytotoxin construct. Examples of well-established non-cleavable linkers include maleimidocaproyl (mc), used with MMAF, and MCC, often used with DM1 conjugates.

## Linker-Calicheamicin Motif

**Table:** 

**Phase**	**Originator**	**Licensee**	**Common name**	**Specificity target**	**Target disease**
3	UCB (Celltech)	Pfizer	CMC-544	anti-CD22	ALL, DLBCL

**Fig. 14:**
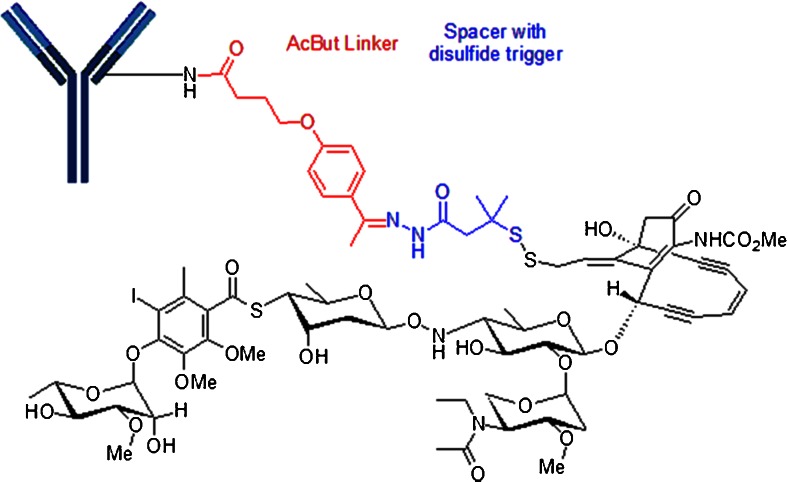
Construct of AcBut-N-Ac-γ-calicheamicin. CMC-544 utilizes an AcBut (4-(4-acetylphenoxy)butanoic acid) linked to an acyl hydrazide derivative of γ-calicheamicin. The acyl hydrazide is produced by the displacement of the methyltrisulfide moiety of γ-calicheamicin with 3-mercapto-3-methylbutyryl hydrazide, termed N-acetyl γ-calicheamicin dimethyl hydrazide.

CMC-544 (Inotuzumab ozogamicin) is in phase 3 clinical trials for acute lymphoblastic leukemia and diffuse large B-cell lymphoma. CMC-544 consists of a humanized IgG4 anti-CD22 antibody covalently linked via an 4-(4-acetylphenoxy)butanoic acid (AcBut) to an acyl hydrazide derivative of γ-calicheamicin as shown in Fig. 14 (29). The acyl hydrazide is produced by the displacement of the methyltrisulfide moiety of γ-calicheamicin with 3-mercapto-3-methylbutyryl hydrazide, termed N-acetyl γ-calicheamicin dimethyl hydrazide. It is worth noting that this linker construct was also utilized in Mylotarg®. Once internalized to the lysosome of the tumor cell, the payload undergoes a two-stage activation process. First, the acid-sensitive hydrazone is hydrolyzed and the aryl tetra saccharide portion of the drug binds to the minor groove of DNA (30). Next is the reduction of the disulfide bond by glutathione, allowing the sulfhydryl intermediate to cyclize onto the enediyne core structure to generate a reactive para-phenylene diradical species that abstracts hydrogen from the phosphodiester backbone to produce double-strand DNA breaks leading to cell death (31).

## Site-Specific Antibody Drug Conjugates

The inherent chemical and biological issues of heterogeneous mixtures have led to efforts directed toward achieving site-specific conjugation. Site-specific conjugation enables the attachment of a specific number of conjugated drugs at defined sites on the antibody, leading to a homogeneous product with well-defined characteristics. The methodologies can be categorized under the heading of 1) genetic engineering of cysteine or seleno cysteine residues, 2) incorporation of non-natural amino acids (nnAA) possessing reactive handles (by either genetic engineering or enzymatic modification) and 3) enzymatic modification (11). These site specific methodologies are listed in Table IV.

**Table IV:** Site-Specific Conjugation Technologies

Description	Methodology	DAR	Conjugation chemistry	Companies	Ref
Engineered Cysteine Residues	Cysteine substitution	2 or 4	Maleimide, Bromoacetamide	Genentech, MedImmune, Seattle Genetics	(32)
Non-natural Amino Acids/Glycans	Seleno cysteine	2	Maleimide	NCI	(33)
	*p*-AcPhe	2 or 4	Oxime	Allozyne, Ambrx	(34)
	FGE (formylglycine generating enzyme)	2 or 4	Oxime, Pictet-Spengler	Catalent Pharma Solutions	(35, 36)
	Azide or alkynyl nnAA or glycan	1 to 2	Click chemistry	Sutro, Allozyne, Synaffix	(37)
Enzymatic	Glycotransferase	2	2-keto-Gal	NCI	(38)
	BTG (bacterial transglutaminase)	2 or 4	Gln with Lys	Innate Pharma Pfizer	http://innate-pharma.com/sites/default/files/iph_poster_world_adc_frankfurt_2014.pdf (39, 40)
	Sortase A	2 or 4	Hydrolysis of Thr-Gly in LPxTG motif	NBE Therapeutics	http://www.nbe-therapeutics.com

**Fig. 15:**
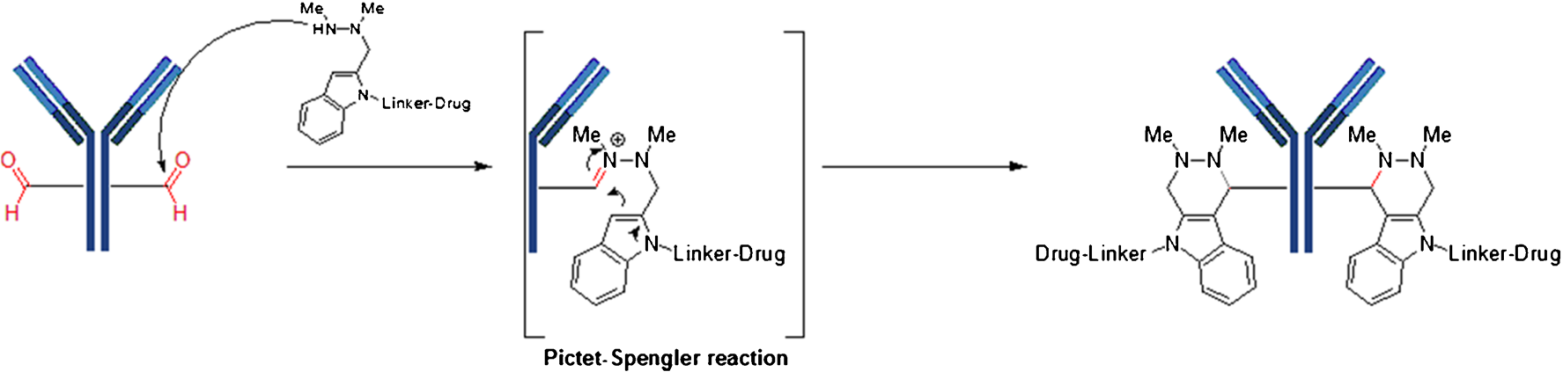
Conjugation based on the production of an aldehyde containing antibody using formylglycine generating enzyme (FGE), an enzyme recognizing the sequence CxPxR and oxidizing the cysteine residue to form formylglycine. The aldehyde is reacted with a cytotoxin-linked indole hydrazine to form a hydrolytically-stable conjugate (Hydrazino-Pictet-Spengler Reaction).

Conjugation through engineered cysteine (or seleno cysteines) utilizes standard cysteine linker chemistry previously described, such as reaction with maleimide or bromoacetamide moieties. However, other non-natural amino acids (nnAA) have been produced via enzymatic modification of the fully constructed mAb. For example, antibodies containing a reactive aldehyde handle have been produced using formylglycine generating enzyme (FGE), an enzyme recognizing the sequence CxPxR and oxidizing the cysteine residue to form formylglycine in the pioneering work of Bertozzi *et al.* (35). The FGE/aldehyde tag technology together with novel bioconjugation chemistry has been termed SMARTag (Specific Modifiable Aldehyde Recombinant Tag) technology. In this technology, the newly generated aldehydes are reacted with indole nucleophiles in a variation of the classical Pictet-Spengler reaction. In the first approach, an indole carrying a hydroxylamine reacts with the aldehyde to produce an intermediate oxyiminium ion which undergoes intramolecular C-C bond formation to form a hydrolytically stable, oxacarboline product (36). The second approach utilizes the hydrazino-Pictet-Spengler (HIPS) reaction to form hydrolytically stable conjugates (Fig. 15). ADCs generated using HIPS chemistry shows >90% stability in human plasma after 7 days at 37°C, regardless of the conjugation site. In addition to producing a very stable C-C bond, the HIPS reaction takes place at neutral or near neutral pH as opposed to the acidic conditions required for typical oxime chemistry. The ability to generate stable bioconjugates using mild, near physiological pH conditions offers numerous advantages over other existing bioconjugation methods (35).

**Fig. 16:**
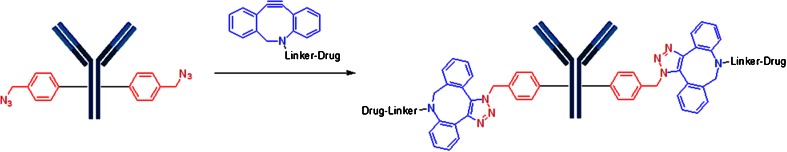
Antibody engineered to contain an non-natural amino acid containing an azido group which can undergo click chemistry with an alkyne-containing linker-drug.

An alternative to enzymatic modification is to use site-specific incorporation of non-natural amino acid (nnAAs) with chemical side chains that are compatible with bio-orthogonal conjugation chemistry. For example, *p*-acetylphenylalanine (pAcPhe), which contains a carbonyl (ketone) as a reactive handle, has been site-specifically incorporated into trastuzumab. The ketone can then react with a drug containing an alkoxy-amine to produce an oxime (34). Other nnAA can be incorporated via translation as the antibody is expressed. This strategy provides controlled sites for conjugation of the cytotoxic drug. A group at Scripps, led by Peter Schultz, has pioneered the encoding of reactive nnAAs proteins by engineering new cell lines or protein expression that have the transcriptional machinery to place a nnAA where it is required (41). An azide containing nnAA, such as para-azidomethyl-L-phenylalanine (pAMF), can be incorporated into the sequence of an antibody, making conjugation via strain-promoted azide-alkyne cycloaddition (SPAAC) copper-free click chemistry a possibility (Fig. 16). Zimmerman, for example, has used this strategy to conjugate monomethylauristatin F (MMAF) to an engineered trastuzamab derivative (37). The resultant ADC displayed high potency in *in vitro* cell cytotoxicity assays.

## Summary

The list of ADCs in the clinic continues to grow, bolstered by the success of ADCETRIS® and Kadcyla®. ADCETRIS® is conjugated to cysteines made available through the reduction of interchain disulfide bridges and contains a protease-sensitive linker, mc-vc-PABC-MMAE, a motif that is utilized in 13 clinical candidates. The additional 11 ADC clinical candidates that conjugate to cysteine utilize maleimide chemistry either to mc or mcc. The remaining 10 candidates with known linker-cytotoxin constructs utilize acylation chemistry to surface lysines of the antibody. Kadcyla® uses a non-cleavable MCC-DM1 (thioether) linker to lysine, a motif that is found in four clinical candidates. An additional five maytansinoid clinical candidates utilize the disulfide linker, SPDB or sulfo-SPDB. The calicheamicin ADC has a AcBut acyl hydrazone-disulfide linker.

The advantage of lysine-based conjugations is that surface lysines are utilized with no alteration of the antibody structure. However, the disadvantage is that there are approximately 30 available lysines for conjugation resulting in ADC heterogeneity. The counter efforts have focused on cysteine conjugation because of its reduced heterogeneity due to the reduction of only 4 interchain disulfides. However, this reduction of the antibody may affect overall ADC-construct stability. In turn, efforts are now focused on the development of bifunctional sulfhydryl linkers that can potentially improve ADC stability.

Significant research efforts are now being directed toward the production of discrete, homogeneous ADC products, via site-specific conjugation. Site-specific conjugation technologies either rely on genetic re-engineering to introduce a discrete, available cysteine or a non-natural amino acid with an orthogonally-reactive functional group handle such as an aldehyde, ketone, azido, or alkynyl tag. Another site-specific conjugation strategy has been selective modification of the antibody by enzymes such as FGE, BTG, sortase, or glycotransferase. These site-specific approaches not only increase the homogeneity of ADCs, but also enable novel bio-orthogonal chemistries that utilize reactive moieties other than thiol from cysteine or amine from lysine. This broadens the diversity of linkers that can be utilized which will lead to better linker design in future generations of ADCs.

## Electronic supplementary material

Below is the link to the electronic supplementary material.

ESM 1 (DOCX 17.8 kb)

## ABBREVIATIONS

AcBut: 4-(4-acetylphenoxy)butanoic acid
ADC: Antibody-drug conjugate
ALL: Acute lymphoblastic leukemia
AML: Acute myeloid leukemia
BTG: Bacterial transglutaminase
CLL: Chronic lymphocytic leukemia
CM: Cyclization module
CRC: Colorectal cancer
DAR: Drug-antibody ratio
DLBCL: Diffuse large B-cell lymphoma
DM1: N^2′^-Deacetyl-N^2′^-(3-mercapto-1-oxopropyl)maytansine
DM4: N^2′^-Deacetyl-N^2′^-(4-mercapto-4-methyl-1-oxopentyl)maytansine
DUBA: Duocarmycin hydroxybenzamide azaindole
FGE: Formylglycine generating enzyme
mc: Maleimidocaproyl
mcc: Maleimidomethyl cyclohexane-1-carboxylate, linked to cysteine of mAb
MCC: Maleimidomethyl cyclohexane-1-carboxylate, linked to thiol of cytotoxin
MDR: Multidrug resistance
MM: Multiple myeloma
MMAE: Monomethyl auristatin E
MMAF: Monomethyl auristatin F
NHL: Non-Hodgkin lymphoma
NHS: N-hydroxy succinimide
nnAA: Non-natural amino acid
NSCLC: Non small-cell lung carcinoma
PABC: Para-aminobenzyloxycarbonyl
PBD: Pyrrolo[2,1-c][1,4]benzodiazepine
RCC: Renal cell carcinoma
SC: Squamous cell
SCCHN: Squamous cell carcinoma of the head and neck
SPDB: N-succinimidyl-4-(2-pyridyldithio)butanoate
sulfo-SPDB: N-succinimidyl-4-(2-pyridyldithio)-2-sulfo butanoate
va: Valine-alanine
vc: Valine-citrulline
