# Supplementary material for: Current ADC Linker Chemistry
Source: Pharm Res. 2015 Mar 11;32(11):3526–40. doi: 10.1007/s11095-015-1657-7 (PMC4596905; doi:10.1007/s11095-015-1657-7)
Supplement: Supplementary file 1 — (DOCX 17.8 kb) [file 11095_2015_1657_MOESM1_ESM.docx]

# Appendix 1. Therapeutic ADCs in Clinical Trials (as of December, 2014)

|  | **Ph** | Originator | Licensee (L)/Collaborator (C) | Intl Nonproprietary Name | Common Name | Specificity Target Name | Target Disease |
| --- | --- | --- | --- | --- | --- | --- | --- |
| 1 | 3 | Seattle Genetics | Celldex (L) | Glembatumumab vedotin | CDX-011 | anti-GPNMB | breast cancer |
| 2 | 3 | UCB (Celltech) | Pfizer | Inotuzumab ozogamicin | CMC-544 | anti-CD22 | ALL,DLBCL, |
| 3 | 2 | Seattle Genetics | Progenics |  |  | anti-PSMA | prostate |
| 4 | 2 | Seattle Genetics | Genentech | Polatuzumab vedotin | RG-7596 | anti-CD79b | NHL |
| 5 | 2 | Seattle Genetics | Genentech | Pinatuzumab vedotin | RG-7593 | anti-CD22 | NHL |
| 6 | 2 | Seattle Genetics | Genentech | lifastuzumab vedotin | RG-7599 | anti-NaPi2b | ovarian cancer |
| 7 | 2 | Immunomedics |  | Labetuzumab-SN-38 | IMMU-130 | anti-CEACAM5 | CRC |
| 8 | 2 | Immunomedics |  |  | IMMU-132 | anti-TROP-2 | solid tumors |
| 9 | 2 | ImmunoGen |  | coltuximab ravtansine | SAR3419 | anti-CD19 | DLBCL |
| 10 | 2 | ImmunoGen |  |  | IMGN 853 | anti-FRα |  |
| 11 | 2 | ImmunoGen | Biotest | indatuximab ravtansine | BT-062 |  | MM |
| 12 | 1 | Seattle Genetics |  | denintuzumab nafodotin | SGN-CD19A | anti-CD19 | ALL & B-cell HNL |
| 13 | 1 | Seattle Genetics |  |  | SGN-CD33A | anti-CD33 | AML |
| 14 | 1 | Seattle Genetics |  |  | SGN-LIV1A | anti-Liv1 | LIV1+ breast cancer |
| 15 | 1 | Seattle Genetics |  |  | SGN-CD70A | anti-CD70 | NHL & RCC |
| 16 | 1 | Seattle Genetics | Agensys(C) |  | ASG-22ME | Nectin-4 | solid tumors |
| 17 | 1 | Seattle Genetics | Agensys(C) |  | ASG-15ME | SLTRK6 | bladder cancer |
| 18 | 1 | Seattle Genetics | Genentech |  | RG-7450 | anti-STEAP1 | prostate |
| 19 | 1 | Seattle Genetics | Genentech | sofituzumab vedotin | RG-7458 | anti-MUC16 | ovarian |
| 20 | 1 | Seattle Genetics | Genentech |  | RG-7882 |  | pancreatic & ovarian |
| 21 | 1 | Seattle Genetics | Genentech |  | RG-7841 |  | solid tumors |
| 22 | 1 | Agensys |  |  | AGS-16M8F/16C3F | anti-AGS-16 | RCC |
| 23 | 1 | Agensys |  |  | AGS67E | anti-CD37 | NHL, CLL, AML |
| 24 | 1 | Seattle Genetics | Takeda |  | MLN-0264 | anti-GCC | gastrointestinal cancer |
| 25 | 1 | Seattle Genetics | Pfizer |  | PF-06263507 | anti-5T4 | solid tumors |
| 26 | 1 | Seattle Genetics | Abbvie |  | ABT-414 | anti-EGFR | SC tumors & glioblastoma |
| 27 | 1 | Seattle Genetics | Abbvie |  | ABBV-399 |  | solid tumors |
| 28 | 1 | Seattle Genetics | Bayer |  | BAY1129980 | anti-C4.4a | solid tumors |
| 29 | 1 | Seattle Genetics | Bayer |  |  | Anti-FGFR2 |  |
| 30 | 1 | Seattle Genetics | GenMab |  | HuMax-TF | anti-TF | solid tumors |
| 31 | 2 | Immunomedics |  | Milatuzumab | IMMU-110/MEDI-115 | anti-CD74 | MM/NHL/  CLL |
| 32 | 1 | ImmunoGen |  |  | IMGN 289 | anti-EGFR | NSCLC, SCCHN, SC NSCLC |
| 33 | 1 | ImmunoGen |  |  | IMGN 529 |  | NHL/CLL |
| 34 | 1 | ImmunoGen | Sanofi |  | SAR 566658 | anti-CA6 | solid tumors |
| 35 | 1 | ImmunoGen | Bayer | anetumab ravtansine | BAY 94-9343 | anti-mesothelin | solid tumors |
| 36 | 1 | ImmunoGen | Amgen |  | AMG 595 | anti-EGFRvIII | glioblastoma |
| 37 | 1 | ImmunoGen | Amgen |  | AMG 172 | anti-CD27L | RCC |
| 38 | 1 | Synthon |  |  | SYD985 | anti-HER2 | HER2^+^ breast cancer |
| 39 | 1 | StemCentRx |  |  | SC16LD6.5 | anti-SCLC | SCLC |
| 40 | 1 | GSK |  |  | GSK2857916 | anti-BCMA | MM |

ALL-acute lymphoblastic leukemia; AML-acute myeloid leukemia; CRC-colorectal cancer; CLL-chronic lymphocytic leukemia; DLBCL-diffuse large B-cell lymphoma; MM-multiple myeloma; NHL- non-Hodgkin lymphoma; NSCLC- non small-cell lung carcinoma; RCC-renal cell carcinoma; SC-squamous cell; SCCHN-squamous cell carcinoma of the head and neck
